# Genome-wide analysis of long non-coding RNAs in sugar beet (*Beta vulgaris* L.) under drought stress

**DOI:** 10.3389/fpls.2023.1118011

**Published:** 2023-02-14

**Authors:** Chunlei Zou, Zhiqiang Guo, Shanshan Zhao, Jishuai Chen, Chunlai Zhang, Haoran Han

**Affiliations:** College of Agriculture, Shanxi Agricultural University, Taigu, China

**Keywords:** drought, *Beta vulgaris* L., long non-coding RNA, target gene, flavonoid biosynthesis, target mimic

## Abstract

Drought stress is one of the most severe abiotic stresses that restrict global crop production. Long non-coding RNAs (lncRNAs) have been proved to play a key role in response to drought stress. However, genome-wide identification and characterization of drought-responsive lncRNAs in sugar beet is still lacking. Thus, the present study focused on analyzing lncRNAs in sugar beet under drought stress. We identified 32017 reliable lncRNAs in sugar beet by strand-specific high-throughput sequencing. A total of 386 differentially expressed lncRNAs (DElncRNAs) were found under drought stress. The most significantly upregulated and downregulated lncRNAs were TCONS_00055787 (upregulated by more than 6000 fold) and TCONS_00038334 (downregulated by more than 18000 fold), respectively. Quantitative real-time PCR results exhibited a high concordance with RNA sequencing data, which conformed that the expression patterns of lncRNAs based on RNA sequencing were highly reliable. In addition, we predicted 2353 and 9041 transcripts that were estimated to be the cis- and trans-target genes of the drought-responsive lncRNAs. As revealed by Gene Ontology (GO) and Kyoto Encyclopedia of Genes and Genomes (KEGG) analysis, the target genes of DElncRNAs were significantly enriched in organelle subcompartment, thylakoid, endopeptidase activity, catalytic activity, developmental process, lipid metabolic process, RNA polymerase activity, transferase activity, flavonoid biosynthesis and several other terms associated with abiotic stress tolerance. Moreover, 42 DElncRNAs were predicted as potential miRNA target mimics. LncRNAs have important effects on plant adaptation to drought conditions through the interaction with protein-encoding genes. The present study leads to greater insights into lncRNA biology and offers candidate regulators for improving the drought tolerance of sugar beet cultivars at the genetic level.

## Introduction

Drought is a most common abiotic stress restricting crop yield and quality in the world. Due to global climate change caused by the greenhouse effect, both the frequency and magnitude of drought are increasing (Naz et al., 2022). Drought stress can induce adverse reactions in plants, such as osmotic imbalance, membrane system damage, and reductions in respiration and the photosynthetic rate, which not only hinder the growth and metabolism of plants at all stages but also reduce the quality and yield of crop (Liu et al., 2020). Drought response in plants is complicated and may occur at the cellular, molecular and physiological levels (Giordano et al., 2016). Similar to other abiotic stress responses, drought response is related to multi-gene interactions within diverse pathways. During drought stress, several regulatory and functional genes undergo differential expression (referred to as differentially expressed genes (DEGs)) in plants, thereby forming complex signaling networks and affecting various biochemical and physiological responses (Wang et al., 2021). For instance, water deficiency can limit plant photosynthetic functions because of decreased leaf expansion, photosynthetic impairment, and decreased Calvin cycle enzyme activities, including phosphoenolpyruvate and Rubisco carboxylase (Bota et al., 2004). Under normal environmental conditions, the production and elimination of reactive oxygen species (ROS) in plant cells are in a dynamic balance. When plants are stimulated by drought stress, this balance is destroyed, resulting in a significant increase of ROS. ROS can target several organelles, like chloroplasts, peroxisomes, and mitochondria. This may result in cell membrane instability, senescence, or plant death (Ma et al., 2013). Hormonal modulation also represents a critical factor that induces water deficiency resistance in plants (Merlaen et al., 2020). Therefore, plants can achieve drought adaption by modulate several pathways, among which noncoding RNAs play essential roles (Chen et al., 2021b).

Noncoding RNAs (ncRNAs) are a class of RNA molecules that do not encode proteins and have catalytic activity, and are widely present in various organisms. Based on structure, ncRNAs can be divided into linear ncRNAs (linear-ncRNAs) and circular ncRNAs (circRNAs). Linear ncRNAs include microRNAs (miRNAs) and long noncoding RNAs (lncRNAs) (Mattick, 2009). LncRNA is a type of ncRNA that is over 200 bp long and plays widespread roles in virtually every plant biological processes (Wang et al., 2018). RNA-sequencing (RNA-seq) is an important approach in transcriptome research, which has helped to identify lncRNAs in several crops and model plants, like *Arabidopsis* (Severing et al., 2018), wheat (Lu et al., 2020), rice (Huang et al., 2021) and *Medicago truncatula* (Pecrix et al., 2018). Particularly, numerous stress-responsive lncRNAs have been identified in rice. They have been suggested to regulate anti-pathogen immunity (Ming et al., 2017) and responses to abiotic stresses like drought (Li et al., 2019a), salinity (Khemka and Singh, 2017), nutrient deficiency (Shin et al., 2018), and heavy metal resistance (Tang et al., 2019).

Numerous lncRNAs acting as endogenous miRNA target mimics (eTMs) have been detected in plants. Typically, lnc_253 and lnc_973 are the possible eTMs for miR156e in cotton under salt stress (Deng et al., 2018). Besides, lncRNA23468 plays the role of ceRNA in tomatoes for regulating NBSLRR genes through the decoy of miR482b with *Phytophthora infestans* (Jiang et al., 2019). LncRNA_1231 is responsible for sequestering miR156b in the flowering period of pigeon pea in a target-mimicry manner, thereby inducing flower-specific SPL-12 transcription factor (TF) up-regulation and modulating flower growth (Das et al., 2020). LncRNA39026 is an eTM for miR168a, which decoys miRNAs for increasing SlAGO1 expression, thereby promoting *Phytophthora infestans* tolerance (Cui et al., 2020). In Cassava, linRNA159 and linRNA340 are the target mimics for miR164 and miR169, respectively, under cold stress (Li et al., 2017). LncRNAs can competitively bind to miR398 while regulating CSD1 expression in winter wheat, thus, improving cold tolerance (Lu et al., 2020). Nonetheless, a few lncRNAs have been characterized experimentally and functionally so far.

Sugar beet (*Beta vulgaris* L.) is an important industrial crop that greatly contributes to the global sugar supply. Sugar beet can acclimate to various abiotic stresses, like salinity, drought, heat, or cold (Zou et al., 2021; Taleghani et al., 2022). In sugar beet, drought acclimation makes it possible to develop drought resistance *via* biochemical/physiological changes (Li et al., 2019b; Alkahtani et al., 2021). Drought response in sugar beet is potentially associated with the expression of protein-coding genes including *BvHb2* (Gisbert et al., 2020). Nonetheless, the roles of lncRNAs in drought-challenged sugar beet remains largely unclear. Therefore, this study aims to explore the role of lncRNAs in sugar beet drought resistance and identify drought-responsive lncRNAs. Therefore, we analyzed lncRNAs from sugar beet leaves under drought condition using high-throughput sequencing technology and bioinformatic approaches. Then the lncRNAs-miRNAs-mRNAs regulatory network related to drought stress response of sugar beet were screened, and quantitative real-time PCR was performed to investigate the expression patterns of drought-related lncRNAs in sugar beet. The results of this study will provide a basis for the next step analysis of the regulatory functions of lncRNAs related to drought resistance in sugar beet.

## Material and methods

### Plant materials and treatments

Sugar beet seeds (KWS9147) were germinated within pots in a greenhouse at 25°C, 80% relative humidity, and 16 h/8 h (day/night) photoperiod. Pot soil was gained from farmland in Taigu, Shanxi. The soil characteristics were as follows: 69.8 mg/kg alkali-hydro nitrogen, 44.7 mg/kg available phosphorus, 244.8 mg/kg available potassium and 15.6 g/kg organic matter.

After emergence, all seedlings were watered to 70% of soil water holding capacity (SWHC) with daily watering, before being subjected to drought stress. At the stage of fully expanding four leaves, the sugar beet seedlings were divided into two groups: (1) well-watered (CK) and (2) drought stress (DR). For the seedlings in the CK group, soil moisture was maintained at 70% of SWHC. Whereas, seedlings in DR group were unwatered for 8 days down to 30% of SWHC, and markedly curled leaves were observed. Then the respective samples were harvested, leaf cuttings were obtained and immediately preserved in liquid nitrogen under -80°C. Ten plants were set in every replicate, and altogether three separate replicates were established.

### Determination of soluble sugar content, peroxidase activity, and salicylic acid content

Leaf soluble sugar content and peroxidase (POD) activity of sugar beet were determined according to the reports of DuBois et al. (2002) and Patra and Mishra (1979), respectively. Salicylic acid (SA) content was measured by the method of Yalpani et al. (1993). Leaves of sugar beet were ground into a fine powder within liquid nitrogen, and approximately 0.50 g samples were collected for subsequent experiments. Methanol was utilized twice for SA extraction. The samples were centrifuged to collect the supernatant, which was divided into two equivalent portions. Thereafter, solvent evaporation till dryness was conducted with the nitrogen stream. One portion was added with 5% trichloroacetic acid (TCA) for SA extraction thrice in the ethyl acetate: cyclopentane: isopropanol (100:99:1, v/v/v). For determining SA, the other portion was added with β-glucosidase (40 U, SIGMA) contained within acetate buffer (0.1 M, pH 5.2), followed by 90-min incubation under 37°C. Later, 5% TCA was added, and SA extract was obtained according to the previous description. Following solvent evaporation, a mobile phase consisting of 0.5 mM EDTA and 0.2 M acetate buffer (pH 5.0) was introduced for dissolving the dry residue, followed by analysis through HPLC and fluorometry using the WATERS Company chromatograph (Milford, Ma, USA) with the 2475 Multi-λ Fluorescence Detector and 2699 Separation Module Alliance. Spherisorb ODS2 WATERS Company column (4.6 × 10 mm, 3 µm) was employed for chromatographic separation. The emission and excitation wavelengths were set at 405 and 295 nm, respectively.

### RNA extraction

Total RNAs were collected from each sample using TRIzol reagent (Invitrogen, Carlsbad, CA, USA), followed by treatment with DNase I (Takara Bio, Dalian, China) to remove contaminating genomic DNA (gDNA). The DNase I-treated total RNA was then incubated with RNase R (3 U/µg, Epicentre, Madison, WI, USA) at 37°C for a 1-h period. Finally, Bioanalyzer 2100 system (Agilent Technologies, CA, USA) and RNA Nano 6000 Assay Kit were employed for assessing RNA quantity and purity. After passing the quality detection, RNAs were used to construct libraries.

### Library construction and RNA sequencing

Total RNA (1 µg/sample) served as input material for preparing the lncRNA library. NEBNext^®^Multiplex Small RNA Library Prep Set for Illumina^®^ (NEB, USA) was utilized for obtaining strand-specific libraries following specific protocols. Later, index codes were added for attributing the sequences to diverse samples. After removing ribosome RNA (rRNA), total RNA was fragmented with divalent cations in NEBNext First Strand Synthesis Reaction Buffer (5X) by increasing temperature. M-MuLV Reverse Transcriptase (RNase H-) and random hexamer primers were used to prepare first-strand cDNA, while RNase H and DNA Polymerase I were utilized for synthesizing second-strand cDNA. Here, dUTP was used rather than dTTP. The rest overhangs were later transfected in blunt ends by polymerase/exonuclease activities. Following the 3’-end adenylation of DNA fragments, they were hybridized through ligation by NEBNext Adaptor containing the hairpin loop structure. 250-300 bp cDNA fragments were selected, and the AMPure XP system (Beckman Coulter, Beverly, USA) was used for purifying the library fragments. After adaptor ligation and size screening, second-strand cDNA was digested with USER Enzyme (NEB, USA) for a 15-min period at 37°C and later for a 5-min period at 95°C before PCR. Index (X) Primer, Universal PCR primers, and Phusion High-Fidelity DNA polymerase were used for the PCR. When the libraries were constructed, they were first quantified *via* Qubit2.0 Fluorometer, followed by dilution till 1.5 ng/µl as well as by detection of library insert size with Agilent 2100 bioanalyzer. When an expected insert size was obtained, we conducted qRT-PCR for the accurate quantification of effective library content (>2 nM) to ensure library quality. For clustering from libraries of acceptable quality, the TruSeq PE Cluster Kit V3-cBot- HS (Illumina) was used. Finally, the Illumina Novaseq 6000 was used for sequencing (Novogene Co., Ltd, China).

### Identification of lncRNA

Raw reads (fastq format) processing was accomplished by adopting in-house Perl scripts. Thereafter, reads containing poly-N, adapter, or those of low quality, were eliminated for obtaining clean data (clean reads). Meanwhile, Q20, Q30, and GC content for all the raw data were determined, followed by the selection of a specific length range for downstream analyses.

The original sequencing data’s clean reads were checked for quality control and compared to the reference genome sequence using HISAT2 (Kim et al., 2015). The genome of *Beta vulgaris* L. RefBeet-1.2.2 (ensemble, release-54) was used as the reference genome (http://plants.ensembl.org/Beta_vulgaris/Info/Index ). StringTie software was used to assemble the results for comparison and to obtain mapped reads (Pertea et al., 2015). To look for unannotated transcription regions, the mapped reads were spliced together and compared to the original genome annotations. Sequences encoding short peptides with fewer than 50 amino acid residues or containing only a single exon were discarded in order to find new transcripts and genes for the species.

The identification of lncRNAs was divided into two stages: basic screening and potential coding-ability screening. The basic screening procedure included selecting (1) transcripts with class_code of “i”,”x”, “u”, “o”, and “e”; (2) transcripts with ≥ 200-bp length and exon numbers ≥ 2; and (3) transcripts with FPKM ≥ 0.1. We used four different software programs designed to screen for coding ability: the CPC, CNCI, and PFAM protein domain analysis tools. We created a Venn diagram to visually display the results of the three analyses, and the same data identified within the three datasets was used for subsequent lncRNA analysis.

### Analysis of differentially expressed lncRNAs (DElncRNAs) and mRNAs (DEmRNAs)

Fold change (FC) and P-values are the screening criteria used to declare differential expression in the process of detecting differential-expression genes. FC is the ratio of expression levels between two samples (groups), and FC and *P*-value are calculated using R-packet DESeq. When screening lncRNA, the standard values that indicate significance are |log2 (FC)|≥ 2 and *P* < 0.05. The screening criteria for mRNA were |log2 (FC)|≥ 2 and FDR < 0.05. (we also adjusted mRNA data based on a false discovery rate [FDR]). String Tie was used to calculate the FPKM (fragments per kilobase of transcript per million fragments mapped) value to assess lncRNA and mRNA expression levels (Trapnell et al., 2010). The differentially expressed lncRNAs and mRNAs were then analyzed using hierarchical cluster analysis.

### Prediction and functional annotations of the target genes of DElncRNAs

The positional relationship between lncRNA and target genes is used to predict lncRNA cis-target genes. Using a Perl script, we identified adjacent genes within 100 kb upstream and downstream of lncRNA as cis-target genes (Zhang et al., 2020). Trans-target genes interact with lncRNA *via* base complementary pairing, and they were predicted using lncTar, a target gene prediction tool (Li et al., 2015).

We used the R package topGO (Ashburner et al., 2000) to perform GO analysis of lncRNA target genes (http://www.geneontology.org/ ) for functional annotation of differential genes. The KOBAS software was used to determine the level of statistical enrichment of lncRNA target genes in the KEGG pathway (Mao et al., 2005).

### Quantitative real-time PCR (qRT-PCR)

By using M-MLV Reverse Transcription Kit (Takara, Dalian, China), we collected total RNA for preparing cDNA through reverse transcription. **Supplementary Table S1** explains the primers utilized (Sangon Biotechnology, Shanghai, China). miRcute SYBR Green MasterMix and SYBR GreenMaster Mix (Tiangen, Beijing, China) were adopted for qRT-PCR that was carried out using LineGene 9600 Plus qRT-PCR detection system (BIOER., Hangzhou, China). 2^−ΔΔCt^ approach was used in data calculation relative to the housekeeping gene of sugar beet *Actin*. The qRT-PCR assay was carried out thrice, and the data were represented as mean ± standard error (SE).

### miRNA target mimicry prediction

Because lncRNAs can act as miRNA target mimics. The lncRNA sequences were submitted to TargetFinder and psRobot software for matching against the sugar beet miRNA database in order to identify lncRNAs that are targeted by miRNAs based on free energy and score value (Fahlgren and Carrington, 2010; Wu et al., 2012).

### ceRNA network construction and analysis

CeRNAs were screened by the ceRNA-score principle (Das et al., 2014; Shu et al., 2019). The criteria were lncRNAs and mRNAs with the same numbers of miRNAs greater than three, *p* < 0.05, and FDR value < 0.1. FDR was calculated by correcting *p*-values using the p.adjust function in R. Then, all of the differentially-expressed lncRNA-miRNA and miRNA-mRNA relationship pairs were gained. Finally, the relationship pairs with negative correlation between miRNAs and their targets and positive correlation between miRNAs targets were selected, and these relationship pairs were combined to obtain ceRNA network. The Cytoscape 3.9.1 software was used to visualize the data.

## Results

### Differences in morphological parameters between two treatments

**Figure 1:**
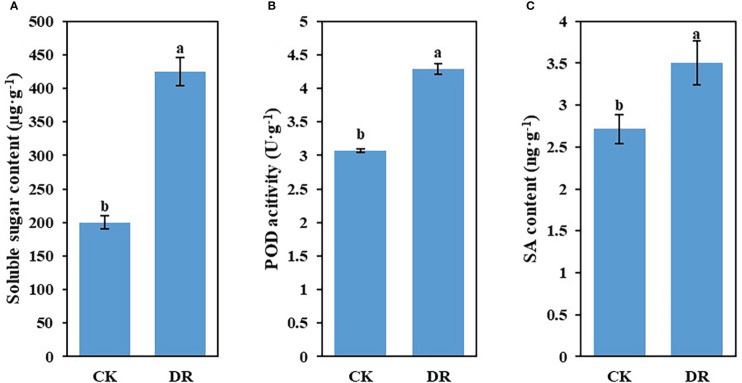
Effect of drought stress on the physiological characteristics of sugar beet. **(A)** Soluble sugar level. **(B)** POD activity. **(C)** SA level. Results are represented by means ± SE.

To examine the impacts of drought stress on the physiological characteristics of sugar beet, we measured soluble sugar content, POD activity, and SA content in leaves harvested in CK and DR-treated plants. As indicated in **Figures 1A–C**, drought stress significantly raised the above-mentioned physiological features. Based on such physiological differences, we contend that there were great differences at the genetic level, including lncRNAs and mRNAs in drought-challenged sugar beet.

### Basic data statistics

Six cDNA libraries (CK1, CK2, CK3 and DR1, DR2, DR3) were constructed. Overall, 93775816, 107421550, 97896732, 106448750, 88929524, and 90913022 raw reads were obtained in CK1, CK2, CK3, DR1, DR2, and DR3 libraries, respectively (**Table 1**). After trimming adapters and filtering out low quality reads, 92758786 (CK1), 106281556 (CK2), 96751372 (CK3), 105457050 (DR1), 88200802 (DR2) and 89932544 (DR3) clean reads were remained and used for further analysis. The Q20 values of clean reads were greater than 96%, the Q30 values of all samples were greater than 89%, and the GC contents were greater than 40%. This suggested that the clean reads were of high quality. We mapped a total of 66308676, 74969665, 69822100, 79273072, 58164384, and 65396407 clean reads from CK1, CK2, CK3, DR1, DR2, and DR3 libraries, respectively, against the reference genome.

**Table 1:** RNA-seq data of six samples.

Samples	CK1	CK2	CK3	DR1	DR2	DR3
Raw reads	93775816	107421550	97896732	106448750	88929524	90913022
Clean reads	92758786	106281556	96751372	105457050	88200802	89932544
Raw bases (G)	14.07	16.11	14.68	15.97	13.34	13.64
Clean bases (G)	13.91	15.94	14.51	15.82	13.23	13.49
Error rate (%)	0.03	0.03	0.03	0.03	0.03	0.03
Q20 (%)	96.97	96.61	96.92	97.07	96.34	96.73
Q30 (%)	91.61	90.87	91.40	91.86	89.96	91.00
GC content (%)	40.88	40.82	41.00	40.21	41.14	40.99
Total mapped reads	66308676 (71.49%)	74969665 (70.54%)	69822100 (72.17%)	79273072 (75.17%)	58164384 (65.95%)	65396407 (72.72%)

### LncRNAs identification in sugar beet

**Figure 2:**
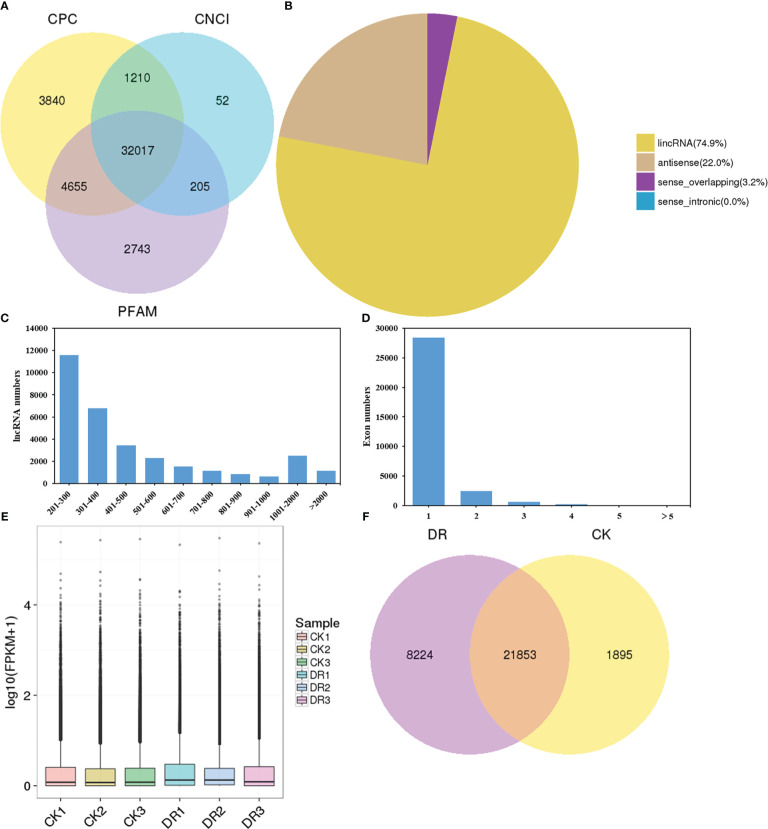
Prime features of lncRNA. **(A)** Venn diagram plot based on CPC, PFAM, and CNCI analyses. **(B)** Diagram showing lncRNA categories. **(C)** lncRNA length distribution. **(D)** Distribution of exon numbers in lncRNAs. **(E)** lncRNAs levels. **(F)** Venn diagram presenting shared and unique lncRNAs under CK and DR conditions.

LncRNAs were screened by complementary base pairing, while the non-coding transcripts were screened by CPC, PFAM, and CNCI. Altogether, 44722 lncRNAs were discovered from the six cDNA libraries, including 41722 from CPC, 39620 from PFAM, and 33484 from CNCI (**Figure 2A**). Each of the three methods identified the same 32017 lncRNAs and so these were considered as novel lncRNAs. Among these novel lncRNAs, 74.9% were located in intergene regions, so we classified them as lincRNA. They occupied a dominant proportion in the novel lncRNAs. Sense-lncRNAs and antisense-lncRNAs, respectively occupied 3.2% and 22.0% of the new lncRNAs (**Figure 2B**). Most lncRNAs (88.5%) had short-length (< 1000 nucleotides, nt), 7.9% had medium-length (1000-2000 nt), while only 3.6% had long-length (> 2000 nt) (**Figure 2C**). As indicated in **Figure 2D**, 96.7% of lncRNAs contained less than two exons. Typically, lncRNAs were associated with the features of small exon number and short transcript length. The box plot displayed the overall distribution of lncRNAs expression levels, of which most had FPKM values between 1 to 10 (**Figure 2E**). As shown in **Figure 2F**, 21853 lncRNAs were simultaneously found in both CK and DR groups, while 8224 and 1895 lncRNAs were uniquely found in CK and DR groups, respectively.

### Analysis of drought-responsive lncRNAs and mRNAs

**Figure 3:**
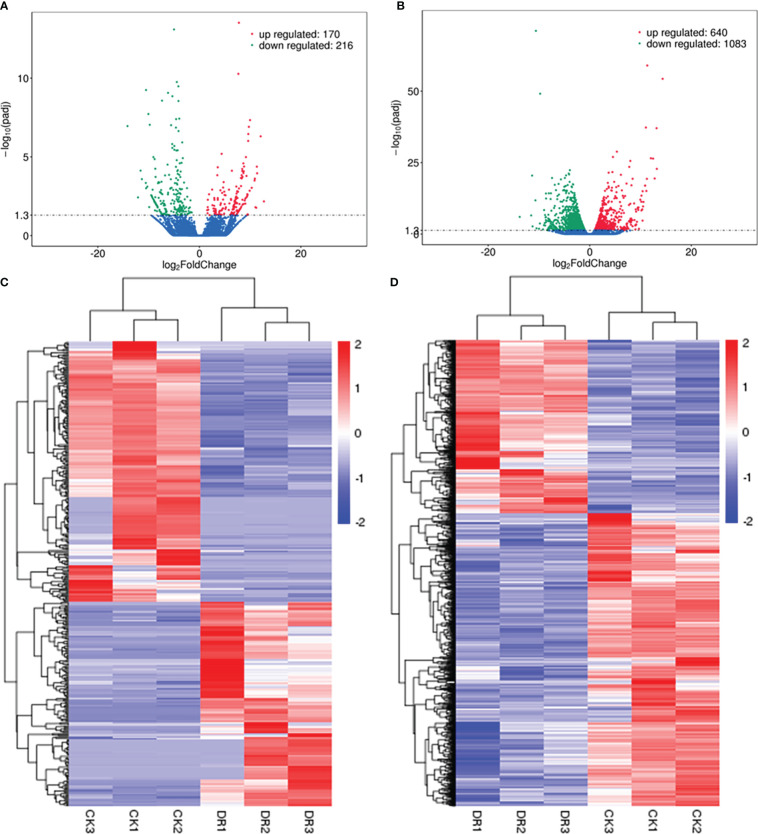
Analysis of DElncRNAs. **(A)** Volcano plot showing DElncRNAs. **(B)** Volcano plot showing DEmRNAs. **(C)** Heatmap for the DElncRNAs. **(D)** Heatmap for the DEmRNAs.

As revealed by sequencing analysis, lncRNAs conforming to *p* < 0.05 and log_2_ (foldchange, FC) ≥ 1 showed significant up-regulation. In contrast, those conforming to *p* < 0.05 and log_2_ (FC) ≤ –1 showed significant down-regulation. Based on the statistical analyses, there were altogether 386 drought-responsive DElncRNAs, including 170 with significant up-regulation and 216 with down-regulation (**Figure 3A**). The clustering analysis for DElncRNAs was displayed in **Figure 3C**. The most significantly upregulated lncRNA was TCONS_00055787 (upregulated by more than 6000 fold), followed by TCONS_00103111 and TCONS_00085517. Conversely, the most significantly downregulated lncRNA was TCONS_00038334 (downregulated by more than 18000 fold), followed by TCONS_00004463 and TCONS_00080695 (**Supplementary Table S2**). Furthermore, 1723 DEmRNAs (**Figures 3B, D**) (640 upregulated and 1083 downregulated) were identified in the DR group compared to the CK group.

**Figure 4:**
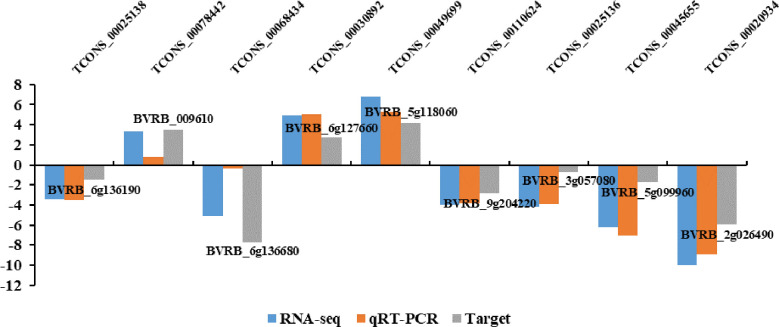
qRT-PCR verification for the nine lncRNAs and their target genes.

To confirm lncRNA expression levels based on RNA-seq, qRT-PCR was conducted to analyze levels of nine random DElncRNAs. Based on the sequencing, these lncRNAs included six down-regulated (TCONS_00025138, TCONS_00068434, TCONS_00110624, TCONS_00025136, TCONS_00045655 and TCONS_00020934) and three up-regulated (TCONS_00078442, TCONS_00030892 and TCONS_00049699) lncRNAs. As a result, the expression patterns of lncRNAs detected by qRT-PCR were highly consistent with those based on RNA sequencing (**Figure 4**), indicating high reliability of lncRNA expression profiles obtained by RNA-seq.

### Predicted target genes for drought-responsive lncRNAs

LncRNA can regulate the expression of its adjacent genes and also act on further genes through base complementary pairing. We predicted 2532 lncRNA cis-target genes within 100 kb upstream and downstream of 386 DElncRNAs (**Supplementary Table S3**). According to the principle of base complementary pairing, we predicted 9041 lncRNA trans-target genes (**Supplementary Table S4**). Concerning the target gene numbers in each lncRNA, many lncRNAs contained multiple target genes. For instance, TCONS_00068434 had five cis-target genes (*BVRB_7g163400*, *BVRB_7g163410*, *BVRB_7g163380*, *BVRB_7g163390*, and *BVRB_7g163420*) and 322 trans-target genes (including *BVRB_6g136680*, *BVRB_2g028320*, and *BVRB_2g039860*). As shown in **Figure 4**, TCONS_00025138, TCONS_00068434, TCONS_00110624, TCONS_00025136, TCONS_00045655, TCONS_00020934,TCONS_00078442, TCONS_00030892, and TCONS_00049699 shared an identical trend of expression with corresponding target genes *BVRB_6g136190*, *BVRB_009610*, *BVRB_6g136680*, *BVRB_6g127660*, *BVRB_5g118060*, *BVRB_9g204220*, *BVRB_3g057080*, *BVRB_5g099960*, and *BVRB_2g026490*, respectively. Moreover, many of these target genes were predicted to encode a variety of proteins, like squamosa promoter-binding-like protein 6 (*BVRB_6 g136190*, targeted by TCONS_00025138), 1-aminocyclopropane-1-carboxylate oxidase 1 (*BVRB_9g204220*, targeted by TCONS_00110624) and receptor-like protein EIX2 (*BVRB_3g057080*, targeted by TCONS_00110811). Our above findings indicated that DElncRNAs might regulate different biological processes to adapt to drought conditions.

### GO and KEGG analyses for potential target genes of DElncRNAs

**Figure 5:**
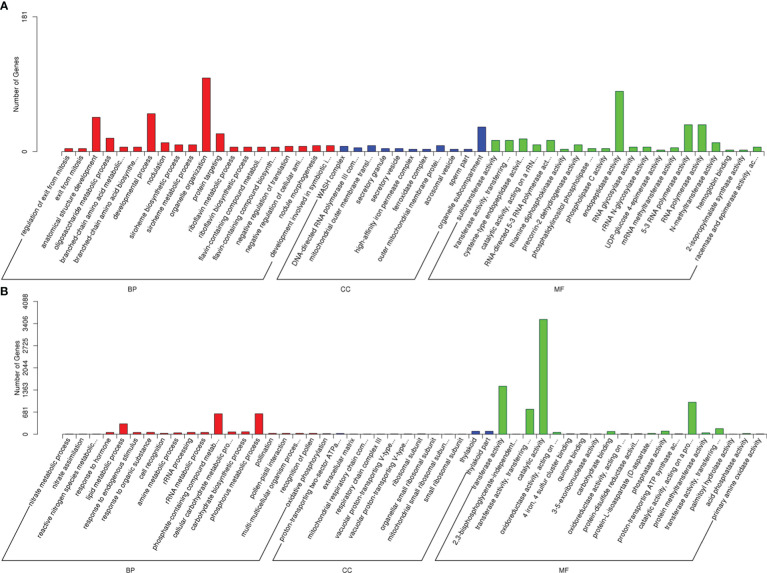
GO classification for the predicted target genes of DElncRNAs. **(A)** Go terms for cis-target genes. **(B)** Go terms for trans-target genes.

For delineating the biological functions, the predicted target genes of the drought-responsive lncRNAs were sorted into GO term categories. As a result, cis-target genes were categorized into 1663, 375 and 812 GO terms in biological process, cellular component and molecular function, respectively (**Supplementary Table S5**), whereas trans-target genes were clustered into 2303, 546 and 1225 GO terms in biological process, cellular component and molecular function, respectively (**Supplementary Table S6**). For the BP category, organelle organization and phosphorus metabolic process were the most highly represented terms for cis- and trans-target genes, respectively, suggesting some functional role of cell organs and phosphorin in drought response (**Figure 5**). In the CC category, cis-target genes related to the organelle sub-compartment, and trans-target genes corresponding to the thylakoid and thylakoid parts were most abundant. Endopeptidase activity for cis-target genes and catalytic activity for trans-target genes were most significantly enriched in the MF category, which indicated that enzymatic reaction played an important role in sugar beet drought response. Meanwhile, the target genes were also found to be enriched in other stress related terms, including developmental process, lipid metabolic process, RNA polymerase activity, and transferase activity.

**Figure 6:**
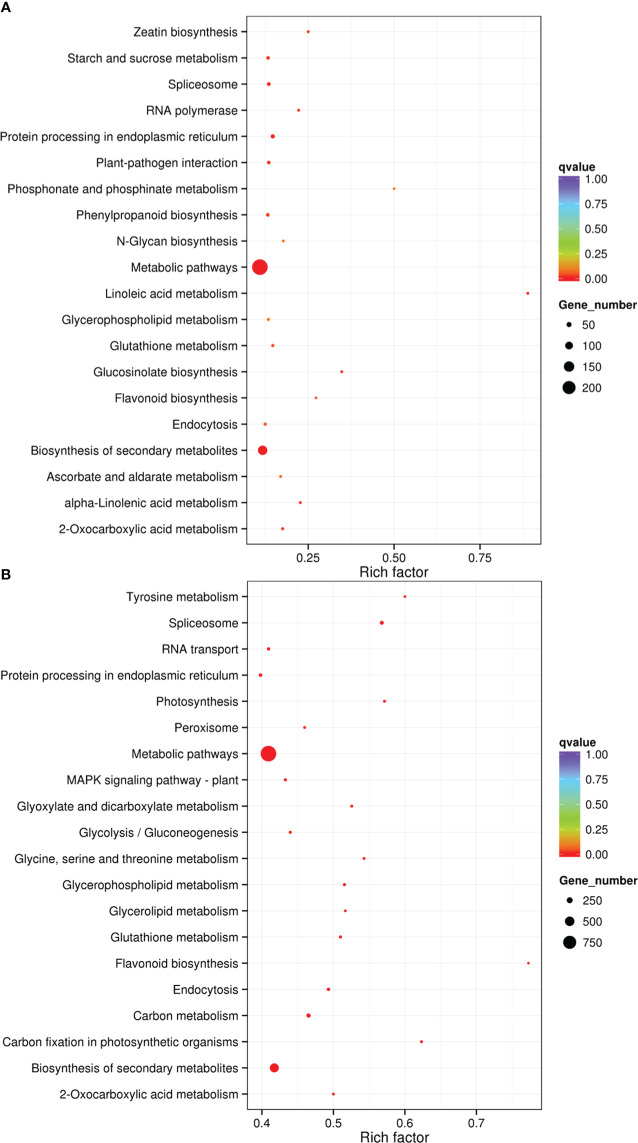
KEGG analysis of the predicted target genes for DElncRNAs. **(A)** KEGG pathways for cis- target genes. **(B)** KEGG pathways for trans-target genes.

The results of KEGG analysis for cis- and trans-target genes for drought-responsive lncRNAs were presented in **Supplementary Tables S7** and**S8**. And the 20 most significantly enriched KEGG pathways were displayed in **Figure 6**. The top four KEGG pathways included linoleic acid metabolism, glucosinolate biosynthesis, flavonoid biosynthesis, and zeatin biosynthesis for cis-target genes (**Supplementary Table S7**; **Figure 6A**), while for trans-target genes, these were carbon fixation in photosynthetic organisms, flavonoid biosynthesis, tyrosine metabolism, and photosynthesis (**Supplementary Table S7**; **Figure 6B**). Among them, the most heavily enriched pathways were linoleic acid metabolism and flavonoid biosynthesis (**Figure 6**). Our observations suggested that these processes play critical roles in drought response. Furthermore, carbon fixation in photosynthetic organisms and photosynthesis were significantly affected when sugar beet plants were subjected to drought condition, suggesting that drought stress significantly inhibited photosynthetic carbon assimilation in sugar beet plants. Flavonoid was found to be a significant determinant of drought tolerance in Arabidopsis (Li et al., 2022). Similarly, our findings also indicated that the application of flavonoid biosynthesis may help to alleviate drought stress in sugar beet plants.

**Figure 7:**
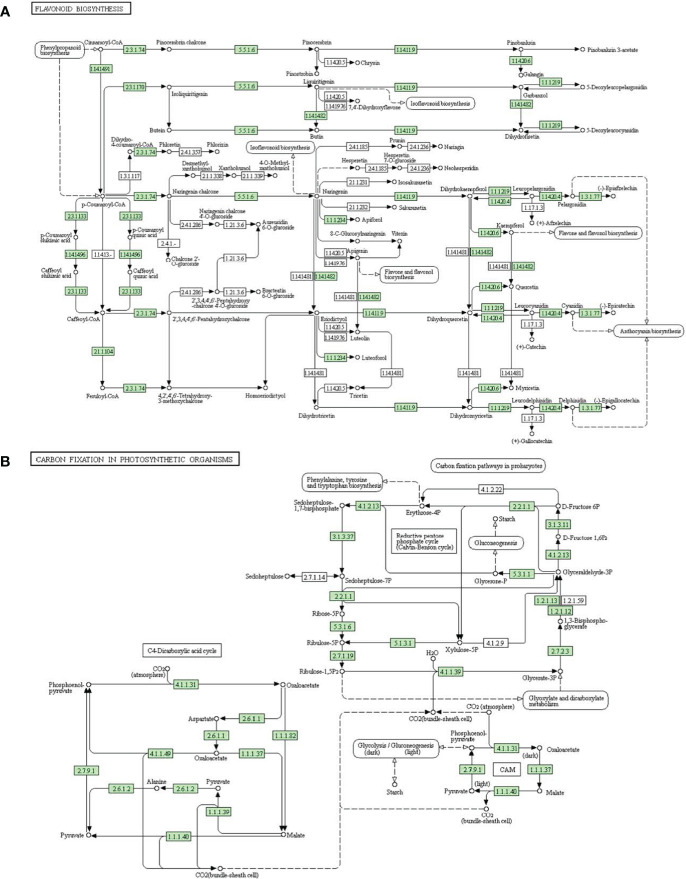
Targets of DElncRNAs in two enriched pathways. **(A)** Flavonoid biosynthesis pathway. **(B)** Carbon fixation in photosynthetic organisms pathway.

Flavonoids are the important elements in abiotic stress resistance in plants (Kovinich and Durkin, 2018; Shah and Smith, 2020; Li et al., 2022). Flavonoid biosynthesis pathway is illustrated in **Figure 7A**. In this study, we identified two genes associated with the flavonoid biosynthesis pathway targeted by drought-responsive lncRNAs: *BVRB_1g007170* encoding dihydroflavonol 4-reductase (EC1.1.1.219) (targeted by TCONS_00009457), and *BVRB_1g016280* encoding flavanone 3-dioxygenase (EC1.14.11.9) (targeted by TCONS_00088109). Chalcone synthase represents a critical enzyme for flavonoid synthesis (Chen et al., 2021c). The two genes, *BVRB_6g151690* and *BVRB_6g152260* encoding chalcone synthase, were estimated as the targets of TCONS_00055970 and TCONS_00056083, respectively.These results indicated that the target genes of DElncRNAs may play critical roles in the biosynthesis of flavonoids during drought stress in sugar beet.

Photosynthetic carbon fixation provides mass and energy sources for various life activities in plants, including stress resistance. In the present study, we predicted several target genes of DElncRNAs related to carbon fixation in photosynthetic organisms (**Figure 7B**). Fructose-1,6-bisphosphatase plays a key role in gluconeogenesis and photosynthetic assimilate sucrose synthesis (Andrew et al., 2017). In our present study, both *BVRB_1g011460* and *BVRB_5g111310*, targeted by TCONS_00057113, encoded fructose-1,6-bisphosphatase (EC3.1.3.37). Malate dehydrogenase is involved in many critical metabolic processes in plants, like glycolysis and photosynthesis (Eprintsev and Gataullina, 2018). Three malate dehydrogenase (1.1.1.37) genes *BVRB_5g124340*, *BVRB_3g049020* and *BVRB_6g137210* were targeted by TCONS_00072169, TCONS_00034128 and TCONS_00002455, respectively. These results suggested that photosynthetic carbon assimilation was critically restrained under drought conditions in sugar beet plants.

### Functional prediction of DElncRNAs acting as miRNA target mimics

LncRNAs can also act as miRNA mimics, disrupting miRNA regulation. Like miRNA-mRNA interactions, lncRNA can be degraded by the corresponding miRNA to reduce its effect on the target mRNA. We used Target Finder and psRobot to predict lncRNAs that could be miRNA target mimics based on free energy, score values, and other filtering conditions. In this study, 1756 distinct lncRNAs were predicted to be potential target mimics of 45 miRNAs, with 42 of those 1756 lncRNAs being DElncRNAs (**Supplementary Table S9**). Multiple DElncRNAs can be targeted by a miRNA, and a miRNA can be targeted by multiple DElncRNAs. For example, the miRNA ath-miR167a-5p targeted 5 DElncRNAs (TCONS_00034119, TCONS_00105108, TCONS_00023171, TCONS_00023940, and TCONS_00100201), and the lncRNA TCONS_00001191 was one common target mimicry of two miRNAs (ath-miR164a and ath-miR164c-5p). Therefore, lncRNAs may have a significant impact on miRNA function.

### Construction of ceRNA network related to drought resistance

**Figure 8:**
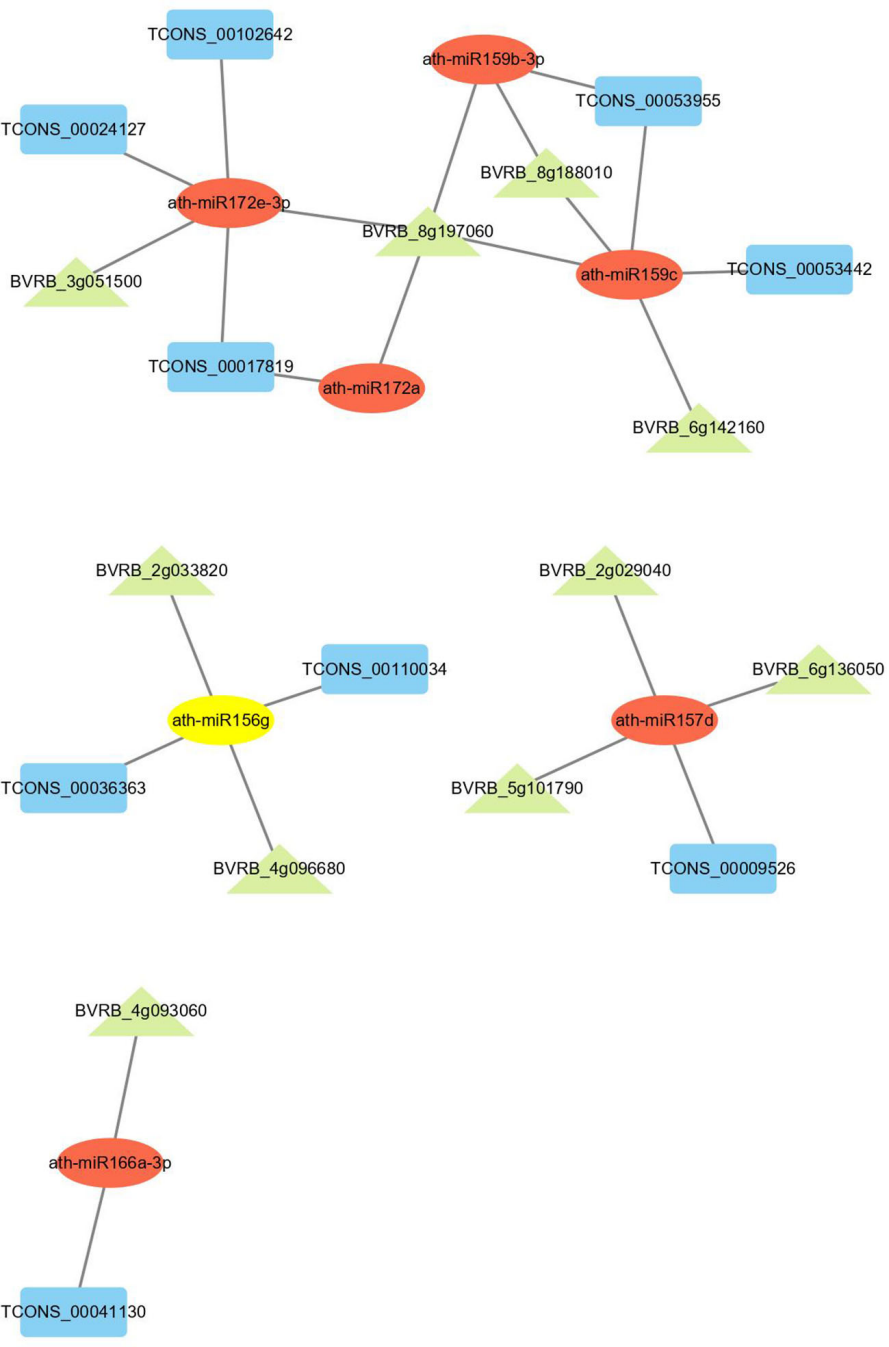
CeRNA network in sugar beet under drought stress. Rectangle, ellipse and triangle nodes represent lncRNAs, miRNAs and mRNAs, respectively. The edges represented the competing interactions among them.

The lncRNA-mRNA-miRNA network was built to evaluate the lncRNA-associated ceRNA interaction landscape in drought-stressed sugar beet. The lncRNA‐mRNA interaction was first discovered using the Pearson correlation coefficient of the expression value. Following that, ceRNAs were filtered using the ceRNA score, which was defined as the number of microRNA response elements (MREs) shared by mRNAs and lncRNAs divided by the total number of lncRNA MREs. Both mRNAs and lncRNAs are targeted for a given lncRNA-miRNA-mRNA triplet. We further filtered the 9 DElncRNAs and 10 DEmRNAs in drought response through literature mining and functional annotation. Based on the selected mRNAs implicated in drought response, we constructed a network of 24 lncRNA-miRNA-mRNA triplets, including 9 lncRNA, 7 miRNAs and 10 mRNAs (**Figure 8**). In this network, one target gene *BVRB_8g197060* was annotated as protein phosphatase 2C and cyclic nucleotide-binding/kinase domain-containing protein based on NCBI nucleotide sequence annotations. *BVRB_6g136050* was annotated as splicing factor-like protein 1. These two target genes with several DElncRNAs were targeted by ath-miR159c and ath-miR157d, respectively.

## Discussion

LncRNA, a type of ncRNA, has been extensively reported in animals, plants and fungi (Zhang et al., 2016). Numerous lncRNAs have been detected in plants; however, their functions remain largely unclear. In particular, the understanding of its role in drought stress is limited. In order to explore the function of lncRNA in drought stress, we used high-throughput sequencing technology to analyze the lncRNA transcriptome in sugar beet cultivar KWS9147 on a genome-wide scale. A total of 32017 lncRNAs were identified in the leaves of sugar beet (**Figure 2F**), which indicated that lncRNA may take part in the regulation of various life activities of sugar beet. Consistent with previous studies (Li et al., 2014; Zhu et al., 2015), most of the novel lncRNAs identified in the present study were located in the intergenic regions (**Figure 2B**), indicating that the type of novel lncRNAs were long intergenic non-coding RNAs (lincRNAs). In addition, lncRNA had short transcript length as well as low exon number (**Figures 2C, D**), which is in line with previous results (Chen et al., 2021a; Xu et al., 2021).

The decreasing water resource has been attributed as the major factor restricting global crop yield and quality. Various responsive mechanisms have evolved in plants to release damage resulting from abiotic stresses (Zhu, 2002). Many protein-encoding genes were found to exert important roles in regulating the response to abiotic stress in plants, like *DREB1A/CBF3* and *SOS1* (Jaglo et al., 1998; Liu et al., 1998; Qiu et al., 2002). Additionally, lncRNAs have been identified as powerful approaches in plants for enhancing their abiotic stress tolerance (Liu and Zhu, 2014). Liu et al. (2012) identified 6484 lincRNAs, among which 1832 responded to salinity, cold, drought, and abscisic acid. Wang et al. (2017) identified 10,785 lncRNAs in legume model species *Medicago truncatula*, including 224 responsive to phosphate deficiency in roots and 358 in leaves. So far, lncRNAs related to drought-responsive modulation were extensively investigated in maize (Zhang et al., 2014), cotton (Lu et al., 2016), *Arabidopsis* (Qin et al., 2017), cultivated rice (Yuan et al., 2018), cassava (Li et al., 2017), and wheat (Cagirici et al., 2017), suggesting a universal role of lncRNAs in drought response in diverse plant species. In the present study, we detected specific and common lncRNAs in control and drought-treated samples of sugar beet for investigating the potential lncRNA functions during drought stress (**Figure 2F**). Compared with the control group, 386 DElncRNAs were found in sugar beet under drought stress, of which 170 were up-regulated and 216 were down-regulated (**Figure 3A**). Moreover, The most significantly upregulated lncRNAs TCONS_00055787 was upregulated by more than 6000 fold (**Supplementary Table S2**), which implied that this lncRNA may play a vital role in sugar beet drougt response.

Meanwhile, 2532 and 9041 transcripts were predicted as the candidate cis- and trans-target genes of drought-responsive lncRNAs, respectively (**Supplementary Tables S3** and **S4**). In plants, promoter-binding-like proteins and receptor-like proteins have been reported to be involved in abiotic stress responses (Wei et al., 2015; Thabet et al., 2021; Rahim et al., 2022). In the present study, several candidate target genes encoded various proteins including squamosa promoter-binding-like protein 6 (*BVRB_6g136190*, targeted by TCONS_00025138), 1-aminocyclopropane-1-carboxylate oxidase 1 (*BVRB_9g204220*, targeted by TCONS_00110624) as well as receptor-like protein EIX2 (*BVRB_3g057080*, targeted by TCONS_00110811). These results suggested that the three DElncRNAs (TCONS_00025138, TCONS_00110624 and TCONS_00110811) may exert important roles in drought stress response of sugar beet by regulating the expression of genes encoding promoter-binding-like proteins and receptor-like proteins.

GO enrichment and KEGG pathway analyses of lncRNAs target genes can help us to understand the functions of lncRNAs more effectively (Muhammad et al., 2022). Oxido-reduction plays a key role in plant abiotic stress responses (Ali et al., 2021). Endopeptidase, catalytic, and transferase activity is closely associated with oxido-reduction (Douglas, 2008). According to GO enrichment analysis, the target genes for drought-responsive lncRNAs were significantly enriched in organelle sub-compartments, thylakoids, endopeptidase activity, catalytic activity, developmental process, lipid metabolic process, RNA polymerase activity, and transferase activity (**Figure 5**). This phenomenon indicated that lncRNAs may be involved in drought resistance by modulating target genes controlling oxidoreductase activity, which was in accordance the report of Huang et al. (2014). Plant flavonoids also play important roles in abiotic/biotic stress responses (Baskar et al., 2018). According to KEGG pathway analysis on the target genes for DElncRNAs, the most significantly enriched pathway was involved in flavonoid biosynthesis (**Figure 6**). In the flavonoid biosynthesis pathway, several target genes of drought-responsive lncRNAs were related to dihydroflavonol 4-reductase and flavanone 3-dioxygenase (**Figure 7A**), implying that lncRNAs may participate in regulating flavonoid biosynthesis to relieve the damage to sugar beet seedlings under drought conditions. Photosynthetic carbon metabolism lays a material foundation for various life activities in the plants (Lawlor and Cornic, 2002). In the present study, one of most enriched pathway was photosynthetic carbon fixation (**Figure 6**). In the photosynthetic carbon fixation pathway, some target genes of DElncRNAs encoded fructose-1,6-bisphosphatase and malate dehydrogenase (**Figure 7B**), which are both involved in photosynthesis (Eprintsev and Gataullina, 2018; Andrew et al., 2017). This result proved that lncRNA could enhance the drought tolerance of sugar beet by affecting photosynthetic carbon metabolism.

MiRNAs and lncRNAs are important transcripts in gene regulation, and lncRNAs may interact with miRNAs as precursors or target mimics (Qiu et al., 2019). Chen et al. (2016) identified 9 intergenic lncRNAs as precursors of 11 known miRNAs in poplar under nitrogen deficiency. Deng et al. (2018) speculated that lnc_973 and lnc_253 regulate the expression of ghr-miR399 and ghr-156e in *Gossypium hirsutum* as a target mimic under salt stress. In the present study, 42 DElncRNAs were predicted as target mimics of several miRNAs (**Supplementary Table S9**), suggesting that lncRNAs were also important for miRNA-mediated regulation under drought stress.

Researchers discovered that lncRNA can function as ceRNA to inhibit miRNA function and compete with other miRNA targets (Salmena et al., 2011). There have been no studies on ceRNA in sugar beets so far. We created a ceRNA network for sugar beet in response to drought stress for the first time using transcriptome sequencing data (**Figure 8**). *BVRB_8g197060* was identified as a protein phosphatase 2C and cyclic nucleotide-binding/kinase domain-containing protein in our network, while *BVRB_6g136050* was identified as a splicing factor-like protein 1. These drought-responsive genes, along with several DElncRNAs, formed a ceRNA network that was targeted by miRNAs such as ath-miR159c and ath-miR157d (**Figure 8**). According to the report of Kobi et al. (2010), *SGN-U567133* represents a novel class of miR159 targets in plants, raising the possibility that post-transcriptional regulation by Sl-miR159 is required for normal tomato development. The expression of potato GAMyb-like genes, which are responsible for drought stress, was negatively regulated by Stu-miR159s (Yang et al., 2014). Growth and development are inhibited in drought-stressed plants (Quiroga et al., 2020). MiR157 was associated with the development in some sorts of plants (Zhou et al., 2021). These findings suggested that lncRNAs may regulate growth, development, and stress tolerance of sugar beet under drought conditions by sponging ath-miR159c and ath-mi157d.

To our knowledge, the current work is the first to report the systemic retrieval, characterization and analysis of lncRNAs isolated from drought-challenged sugar beet plants using high-throughput sequencing. From the results of the present study, we conclude that lncRNAs play important roles on sugar beet adaptation to drought conditions through the interaction with protein-encoding genes. Our findings provide newfound information regarding the potential role of lncRNAs in response to drought stress, further research is required to elucidate the molecular mechanisms of significantly dysregulated lncRNAs.

## Data availability statement

The datasets presented in this study can be found in online repositories. The names of the repository/repositories and accession number(s) can be found below: https://www.ncbi.nlm.nih.gov/, GSE205413.

## Author contributions

CZo: Investigation, writing, review and editing. ZG, SZ, and JC: Investigation. CZh: Conceptualization, Writing - review & editing. HH: Investigation. All authors contributed to the article and approved the submitted version.
