# Supplementary material for: Genome-wide analysis of long non-coding RNAs in sugar beet (Beta vulgaris L.) under drought stress
Source: Front Plant Sci. 2023 Feb 14;14:1118011. doi: 10.3389/fpls.2023.1118011 (PMC9971629; doi:10.3389/fpls.2023.1118011)
Supplement: Supplementary file 1 [file Table_1.docx]

**(1)** **Primer sequences of housekeeping gene**

Actin-F AATGTTCCCTGGTATTGCTGAC

Actin-R CACTTTCTGTGGACGATTGATG

**(2) Primer sequences of objective lncRNAs**

TCONS_00025138-F 5' TCATCAATCTTATCAATGTAATCCC 3' 57.5

TCONS_00025138-R 5' CACTTAATGATATTTGTGACGATGA 3' 57.0 97bp

TCONS_00078442-F 5' CATCTATTGCTAACACCCACGA 3' 58.1

TCONS_00078442-R 5' CTGCCAGAACCTCTTTCACTTT 3' 58.2 249bp

TCONS_00068434-F 5' GACTTGTTTGGGTTGCTGGA 3' 58.2

TCONS_00068434-R 5' AGATTCTACAGAAAATTCGGCAG 3' 57.8 119bp

TCONS_00030892-F 5' AAAAGGAATACGGAGTTTGTCG 3' 58.3

TCONS_00030892-R 5' GCTCAGTGGTAGGTGTTGGG 3' 57.6 251bp

TCONS_00049699-F 5' AACGAAAGAGGGACAAAGAGC 3' 58.0

TCONS_00049699-R 5' ACATCTGAAATCATCACGGAAAT 3' 58.0 130bp

TCONS_00110624-F 5' GCCATTATAAGTAAGTTCTAAGGGG 3' 58.1

TCONS_00110624-R 5' AACACATCCTACACACCTATTAACG 3' 57.7 92bp

TCONS_00025136-F 5' TATCCCACATCTTGCCTTTCA 3' 58.1

TCONS_00025136-R 5' GGCCACTATATTTTGACAACACTAC 3' 57.5 184bp

TCONS_00045655-F 5' TCTTGTTTGATTCGCCCAGT 3' 58.0

TCONS_00045655-R 5' TATATCTCGGATTTAGACATGCTTG 3' 58.2 191bp

TCONS_00020934-F 5' GTTTACCGCATCCAAAGTCC 3' 57.2

TCONS_00020934-R 5' CCTACGAGAGTTGTGAGTATGGTT 3' 57.4 113bp

**(3) Primer sequences of partial target genes**

BVRB_6g136190-F 5' CCACTAATCTCAAATACACAAGCAC 3' 58.2

BVRB_6g136190-R 5' TTTCTTCACCAAATGAGGGATAA 3' 58.6 157bp

BVRB_009610-F 5' CAATCAGCCTACACTAACAAACCT 3' 57.8

BVRB_009610-R 5' CAGTTACCTCTGCCTGTCTCTTC 3' 57.7 232bp

BVRB_6g136680-F 5' GCCTACCGTTCAGGAGATGC 3' 59.6

BVRB_6g136680-R 5' GTCCTGATTACTACGGATGGTTTTA 3' 59.2 117bp

BVRB_6g127660-F 5' GCACCTCACTGACTACCCTACAC 3' 58.1

BVRB_6g127660-R 5' TGAAAGCGAAGTTCTACGAGTG 3' 57.8 148bp

BVRB_5g118060-F 5' AACTTGGTTCGCTTCCCG 3' 58.3

BVRB_5g118060-R 5' CTTCCGATCATCAACTCAGTCA 3' 57.5 138bp

BVRB_9g204220-F 5' GCAGAGAAGGATGCAGAGGAC 3' 58.8

BVRB_9g204220-R 5' GCAGCAACAGTGCCCAAG 3' 57.8 158bp

BVRB_3g057080-F 5' TTCAAATGGCAATAATAAGCAAG 3' 57.6

BVRB_3g057080-R 5' CAAACTAACAAGGGAAGAACTAAGA 3' 57.0 259bp

BVRB_5g099960-F 5' ATTGCTACCGATAGTGATGGAGA 3' 58.4

BVRB_5g099960-R 5' CTCTATCCAACAAATCAGGAATCA 3' 58.2 199bp

BVRB_2g026490-F 5' GTTCAAGTAGCCCAAGCCC 3' 57.1

BVRB_2g026490-R 5' TATCAATCCGAAACAACGCTC 3' 58.0 222bp
